# The diversity and specificity of the extracellular proteome in the cellulolytic bacterium *Caldicellulosiruptor bescii* is driven by the nature of the cellulosic growth substrate

**DOI:** 10.1186/s13068-018-1076-1

**Published:** 2018-03-23

**Authors:** Suresh Poudel, Richard J. Giannone, Mirko Basen, Intawat Nookaew, Farris L. Poole, Robert M. Kelly, Michael W. W. Adams, Robert L. Hettich

**Affiliations:** 1 Biosciences Division, Oak Ridge National Laboratory, Oak Ridge, TN 37831 USA; 2 Chemical Sciences Division, Oak Ridge National Laboratory, Oak Ridge, TN 37831 USA; 3 BioEnergy Science Center at Oak Ridge National Laboratory, Oak Ridge, TN 37831 USA; 4 Department of Genome Science and Technology, University of Tennessee, Knoxville, TN 37996 USA; 5 Department of Biochemistry and Molecular Biology, University of Georgia, Athens, GA 30602 USA; 6 Present Address: Department of Molecular Microbiology and Bioenergetics, Institute of Molecular Biosciences, Johann Wolfgang Goethe University, Frankfurt Am Main, Germany; 7 Department of Chemical and Biomolecular Engineering, North Carolina State University, Raleigh, NC 27695 USA; 8 Present Address: Department of Biomedical Informatics, College of Medicine, University of Arkansas for Medical Sciences, Little Rock, AR 72205 USA

**Keywords:** *Caldicellulosiruptor bescii*, Carbohydrate-active enzymes (CAZymes), Extracellular solute binding proteins (ESBPs), Protein of unknown function (PUF), Glycosyl hydrolases (GH), Mass spectrometry, Switchgrass, Xylan, Avicel, C5 substrates, C6 substrates, Lignocellulosic, Extracellular

## Abstract

**Background:**

*Caldicellulosiruptor bescii* is a thermophilic cellulolytic bacterium that efficiently deconstructs lignocellulosic biomass into sugars, which subsequently can be fermented into alcohols, such as ethanol, and other products. Deconstruction of complex substrates by *C. bescii* involves a myriad of highly abundant, substrate-specific extracellular solute binding proteins (ESBPs) and carbohydrate-active enzymes (CAZymes) containing carbohydrate-binding modules (CBMs). Mass spectrometry-based proteomics was employed to investigate how these substrate recognition proteins and enzymes vary as a function of lignocellulosic substrates.

**Results:**

Proteomic analysis revealed several key extracellular proteins that respond specifically to either C5 or C6 mono- and polysaccharides. These include proteins of unknown functions (PUFs), ESBPs, and CAZymes. ESBPs that were previously shown to interact more efficiently with hemicellulose and pectin were detected in high abundance during growth on complex C5 substrates, such as switchgrass and xylan. Some proteins, such as Athe_0614 and Athe_2368, whose functions are not well defined were predicted to be involved in xylan utilization and ABC transport and were significantly more abundant in complex and C5 substrates, respectively. The proteins encoded by the entire glucan degradation locus (GDL; Athe_1857, 1859, 1860, 1865, 1867, and 1866) were highly abundant under all growth conditions, particularly when *C. bescii* was grown on cellobiose, switchgrass, or xylan. In contrast, the glycoside hydrolases Athe_0609 (Pullulanase) and 0610, which both possess CBM20 and a starch binding domain, appear preferential to C5/complex substrate deconstruction. Some PUFs, such as Athe_2463 and 2464, were detected as highly abundant when grown on C5 substrates (xylan and xylose), also suggesting C5-substrate specificity.

**Conclusions:**

This study reveals the protein membership of the *C. bescii* secretome and demonstrates its plasticity based on the complexity (mono-/disaccharides vs. polysaccharides) and type of carbon (C5 vs. C6) available to the microorganism. The presence or increased abundance of extracellular proteins as a response to specific substrates helps to further elucidate *C. bescii*’s utilization and conversion of lignocellulosic biomass to biofuel and other valuable products. This includes improved characterization of extracellular proteins that lack discrete functional roles and are poorly/not annotated.

**Electronic supplementary material:**

The online version of this article (10.1186/s13068-018-1076-1) contains supplementary material, which is available to authorized users.

## Background

Microbial utilization of lignocellulose requires cellulases and xylanases that synergistically unravel and hydrolyze the carbohydrate content of plant biomass within the lignin matrix [1, 2]. Microcrystalline cellulose is the most recalcitrant component, and is most effectively deconstructed with either non-complexed cellulases [3] or self-assembled cellulases/CAZymes on protein scaffolds known as cellulosomes [4]. While the fungal responses to simple and complex C6 and C5 substrates have been studied extensively [3, 5–9], only relatively few comprehensive studies have been performed with cellulolytic bacteria [10, 11]. Some organisms, such as the bacterium *Caldicellulosiruptor bescii* (previously *Anaerocellum thermophilum*), secrete multi-functional cellulases that contain both binding and catalytic domains [10, 12] for this purpose [13]. The cellulose deconstruction mechanism of *C. bescii* is distinct from either that of the fungal free or non-complexed cellulases and Clostridial cellulosomes [14]. *C. bescii* is a Gram-positive, anaerobic, thermophilic bacterium that grows optimally at 78 °C, with a temperature maximum as high as 90 °C, and can ferment crystalline cellulose and xylan as well as untreated plant biomass (including poplar and switchgrass) [15, 16]. These untreated lignocellulosic substrates are attacked primarily by an array of glycoside hydrolases (GHs) that includes several large, multi-domain carbohydrate-active enzymes (CAZymes) [17], as well as other accessory enzymes and substrate binding proteins that are integral to the deconstruction process.

The multi-domain architecture of these complex cellulolytic proteins involves glycosyl hydrolase (GH) domains interspersed with carbohydrate-binding modules (CBMs), which play a major role in localizing the catalytic domain in close proximity to the substrate [18]. The *C. bescii* genome encodes 52 annotated extracellular GHs that aid in the deconstruction of the carbohydrate components of cell walls [19]. CelA has been reported as one of the most abundant enzymes in this group [10], and consists of three CBMs and two catalytic domains (GH9 and GH48) with both endo- and exoglucanase activities [20, 21]. Reduction in exoglucanase (GH48) activity during extracellular deconstruction of substrates was reported when CelA was deleted from the *C. bescii* genome [22]. Functional analysis of the genomic region that includes six major GH coding genes, identified as the glucan degradation locus (GDL), revealed the important roles of glycoside hydrolases in the deconstruction of plant biomass. In particular, the synergistic activity of Athe_1867 (CelA), Athe_1859, and Athe_1857 accounted for deconstruction of 92% of microcellulose (Avicel) [23] and a related study shows similar pattern in vitro [24]. Transcriptomics measurements of the growth of *Caldicellulosiruptor* species on cellulose or switchgrass revealed that many carbohydrate ABC transporters and multi-domain extracellular GHs are differentially regulated [17]; in fact, the expression of as many as 32 GHs responded to growth on microcrystalline cellulose when compared to glucose [25]. A previous proteomics study of the *C. bescii* secretome revealed that the most abundant proteins were multi-domain glycosidases, extracellular solute binding proteins (ESBPs), flagellin, putative pectate lyases, and uncharacterized proteins with predicted secretion signals [10]. However, the study was limited to crystalline cellulose as the only growth substrate.

To date, most studies of *C. bescii* have focused on a limited number of growth conditions [20, 26–29]. As there is no comprehensive study examining *C. bescii*’s growth on a variety of C5- and C6-substrates, the functional roles of loosely characterized or uncharacterized extracellular proteins critical to lignocellulose solubilization remain undefined. To this end, we investigated the extracellular proteome of *C. bescii* grown on six different carbon sources, including substrates that are mono-, di-, and polysaccharide in nature. Glucose and xylose (monosaccharides), along with cellobiose (disaccharide), were selected as “simple substrates,” whereas crystalline cellulose (Avicel), xylan, and switchgrass (polysaccharides) were selected as “complex substrates.” Comparisons between the extracellular proteins measured across substrate classes (i.e., simple vs. complex, C5 vs. C6, pairwise comparisons between substrates) were conducted to ascertain substrate-specific dependencies that both further inform the process of lignocellulosic deconstruction and utilization by *C. bescii* as well as lend additional functional information to poorly characterized proteins involved in the process (i.e., ESBPs that respond to specific substrates or substrate classes). Additionally, a careful examination of “proteins of unknown function” was undertaken to determine which of these are highly growth substrate dependent.

## Methods

### Cultivation and sampling

Medium for the cultivation of *Caldicellulosiruptor bescii* DSM6725 was supplemented with vitamins, trace elements, and 0.5 g L^−1^ yeast extract, as described previously [15]. Medium was initially prepared without the carbon source. Six different growth substrates were picked to represent a range of simple to complex cellulose substrates for differential proteome characterizations. The carbon sources—glucose, cellobiose, crystalline cellulose (Avicel PH-101, Sigma), xylose, birchwood xylan (Sigma) or Switchgrass (sieved 20/80-mesh fraction; BESC Alamo cultivar provided by Dr. Brian Davison, Oak Ridge National Laboratory, Oak Ridge, TN), were then added to the medium. The switchgrass samples were used without chemical or physical pretreatment, other than washing for 18 h with water at 78 °C, and will be referred to as ‘unpretreated switchgrass’ [30]. The bottles containing media and growth substrates were then closed with butyl rubber stoppers, and the headspace was replaced with N_2_^−^/CO_2_ (80/20). Growth experiments were performed at 78 °C as closed cultures without pH control (400 mL volume, shaken at 150 rpm). Growth was monitored by cell counting using a Petroff-Hausser counting chamber. To better provide for relative proteome comparisons across all samples, the bottles for each growth condition were harvested from the incubator at similar growth stage (all were mid-to-late exponential phase) and all at similar cellular densities (0.5–1.5 × 10^8^ cells mL^−1^). The culture was immediately (< 1 min) brought to room temperature by pumping it through a glass cooling coil bathed in an ice-water slurry, as previously described [31]. Most of the insoluble substrate was removed by this procedure. Cells and residual substrate were pelleted by centrifugation at 6000×*g* for 5 min. Subsamples (50 mL) of the supernatant containing the secreted proteins were carefully decanted, frozen, and kept at − 80 °C until further processing.

### Fermentation product analyses

Fermentation products were determined by high-performance liquid chromatography (HPLC) in the culture supernatants after removal of cells and insoluble substrates by centrifugation. Centrifuged samples for HPLC were further acidified with 0.1 M H_2_SO_4_ and centrifuged again before analysis to remove particles. Organic acids, cellobiose, glucose, and xylose were determined using a 2690 separations module (Waters, Milford, MA) equipped with an Aminex HPX-87H column (300 mm by 7.8 mm; Bio-Rad, Hercules, CA), a photodiode array detector (model 996; Waters) and a refractive index detector (model 410; Waters). The system was operated with 5 mM H_2_SO_4_ as the eluent at a flow rate of 0.5 mL min^−1^.

## 2D LC–MS/MS-based proteomic analysis

Cell-free secretome samples were prepared for 2D LC–MS/MS analysis as described previously [10]. Briefly, filter-concentrated supernatant proteins (5 kDa MWCO spin column; Vivaspin20 by Sartorius) were denatured and reduced with SDS lysis buffer plus DTT and subjected to TCA precipitation to enrich proteins and remove bulk SDS and other small molecules. Acetone-washed protein pellets were then resolubilized in urea and concentrations assessed by BCA (Pierce). Recovered supernatant proteins were again reduced with DTT, alkylated with IAA to block disulfide bridge reformation, and digested to peptides with two sequential aliquots of sequencing-grade trypsin (Promega Corp., Madison, WI, USA) at a 1:100 enzyme:protein ratio (w/w), initially overnight then followed by 4 h at room temperature. As previously described, samples were diluted 1:1 prior to overnight digestion, then again before 4-h digestion [10]. Samples were then salted (NaCl), acidified (formic acid), and filtered through a 10-kDa MWCO spin column filter (Vivaspin2; Sartorius). Peptide concentrations were then measured using the BCA (Pierce). Five micrograms of peptides were then pressure-loaded onto a biphasic MudPIT back column, as described previously [10, 32, 33]. Bound peptides were then washed, separated, and analyzed by data-dependent MS/MS over 2 successive salt cuts of ammonium acetate (50 and 500 mM). LC-resolved peptides were analyzed by a ThermoFisher LTQ-Orbitrap-XL mass spectrometer.

### MS data analysis and evaluation

Acquired MS/MS spectra were matched with theoretical tryptic peptides generated from a concatenated *C. bescii* proteome FASTA database with contaminants and decoy sequences using MyriMatch v. 2.1 [34]. Peptide spectral matches (PSM) were filtered to achieve peptide false-discovery rates (FDR) < 1% and assembled to their respective proteins using IDPicker v. 3.0 [35]. Protein abundances were derived via peptide ion intensity values as previously described [36]. Extracellular proteins were analyzed independently by computationally removing PSORT- and Phobius-predicted intracellular proteins, as their presence in the supernatant fraction is largely due to contamination via microbial lysis [37, 38]. Following normalization, pairwise comparisons were conducted between all substrates using Student’s *t* test and resulting *p* values for each protein were adjusted via Benjamini–Hochberg (BH) FDR correction. Comparisons resulting in BH-corrected *p* values < 0.05 were further investigated. Comparisons across broad substrate classes were similarly performed, namely C5 vs. C6 substrates as well as complex vs. simple substrates. Functional enrichment (KEGG and GO) for specific substrates were performed through the R package PIANO [39] as detailed previously [40]. A genome atlas was created that charted the abundance profiles of extracellular proteins across substrates—globally visualized in the context of the entire *C. bescii* chromosome using CMG-botools [41]. All the heat maps were generated using Orange [42]. All raw mass spectra for the proteome measurements have been deposited into the ProteomeXchange repository with the following accession numbers: (MassIVE Accession: MSV000081856, ProteomeXchange: PXD008556, FTP link to files: ftp://MSV000081856@massive.ucsd.edu, Reviewer password: ‘a’).

## Results and discussion

### Substrate-dependent growth state characteristics

**Fig. 1:**
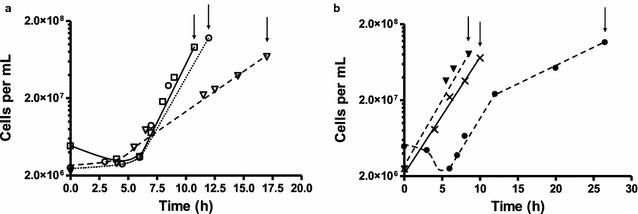
Growth of *C. bescii* on complex medium with different **a** soluble or **b** insoluble components of plant biomass. Cultivation was performed in 1-L closed bottles filled with 400 mL medium, under a N_2_/CO_2_ (80/20) atmosphere, with glucose (open squares), cellobiose (open circles), xylose (open triangles), crystalline cellulose (closed circles), birchwood xylan (closed triangles), and on unpretreated switchgrass (crosses), at substrate concentrations of 5 g L^−1^. After cultures reached the mid-to-late exponential growth phase (7.0–1.5 × 10^8^ mL^−1^; indicated by arrows), supernatants containing the extracellular proteins were separated from the cells by centrifugation. Representative growth curve shown for each substrate

Microbial growth observed on the different substrates was consistent with previous reports [15, 43] (Additional file 1: Table S1). The highest growth rates were achieved with glucose and cellobiose (with doubling times of 0.67 and 0.57 h^−1^, respectively) as shown in Fig. 1. Growth on the C5 xylose was significantly slower (0.24 h^−1^) than the growth on xylan (0.43 h^−1^). This may imply that xylo-oligomers derived from xylan are taken up by *C. bescii* faster than xylose, or that growth can be enhanced by other components of xylan, such as l-arabinose or d-glucuronic acid [44], undoubtedly similar to *C. thermocellum* preference to transport longer oligomers to save ATP requirements [45]. For comparison, a reduced growth rate of thermophiles has been observed with increasing xylose concentrations, starting as low as 2 g L^−1^ [46]. This may be due to an enhanced Maillard reaction with xylose (as compared to glucose) [47], which releases substances inhibitory to microbial growth. For crystalline cellulose, an initial growth rate of 0.37 h^−1^ was observed following a short lag phase—a rate which agrees with bioreactor studies [15, 30]. Similarly, the growth rate on unpretreated switchgrass (0.34 h^−1^) was only slightly lower than what has been reported in pH-controlled reactors. Since the cultures were harvested early, in mid-to-late exponential growth phase, and the studies were performed without pH control [30], only low amounts of products (Additional file 1: Table S1) were found in the supernatant. Among these, acetate was the dominant product, with up to 3.1 mM produced in the xylose fermentations, while lower amounts of lactate < 1 mM were detected. These concentrations agree with what has been published previously for *C. bescii* in the exponential growth phase [43]. Gas phase composition was not determined. Taken together, the observed growth rates and formed products compared well with previous reports and indicated that the cultures were suitable for the analysis of the extracellular proteome. To this end, the culture supernatants were separated from the cells by centrifugation, and proteins in the supernatant were further characterized.

### Overview of the expressed extracellular proteome across divergent substrates

**Fig. 2:**
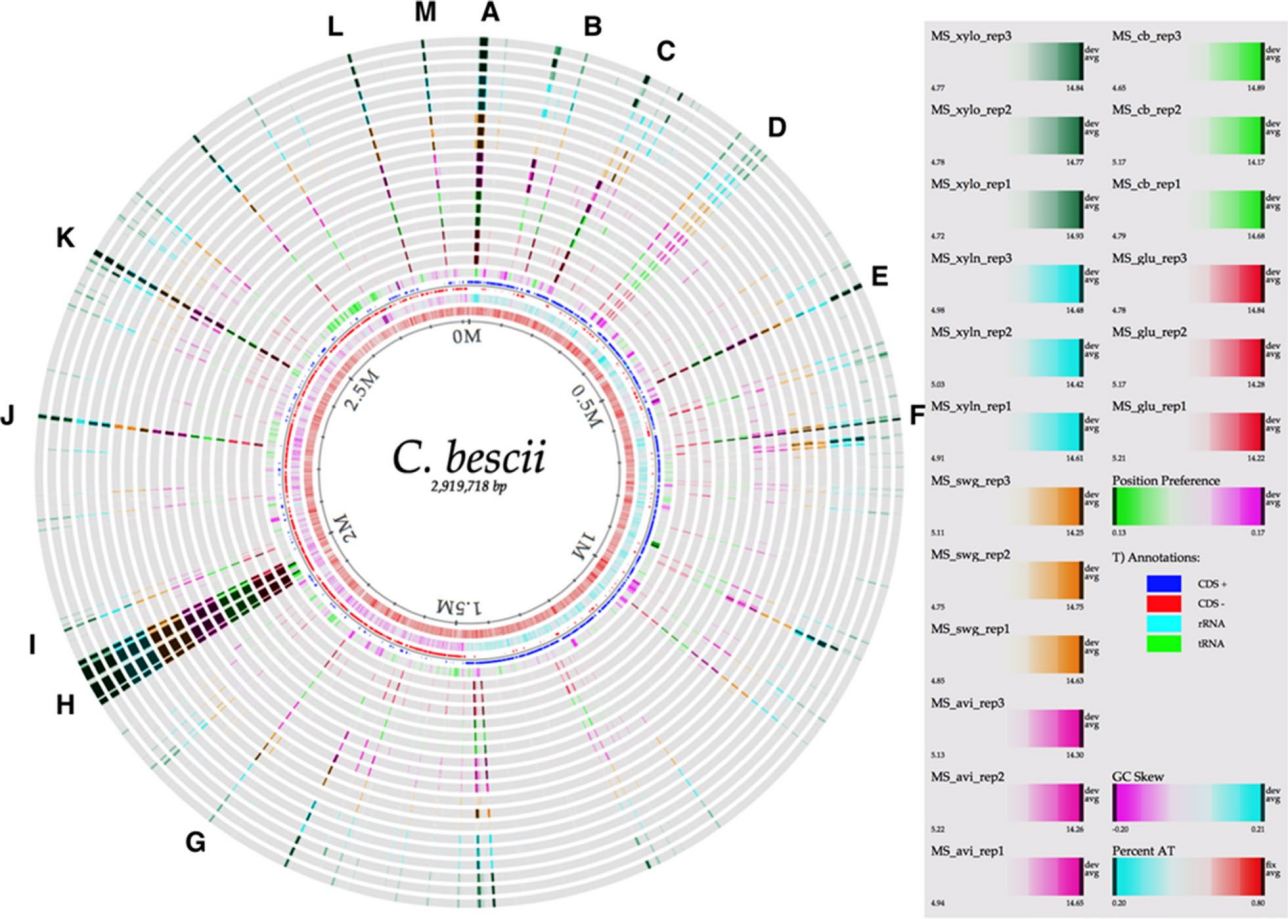
Genome atlas overlaid with abundance profiles of proteome obtained from the mass spectrometry experiments. The innermost circle with bases position is the circular genome. The outer 18 lanes are the biological samples (6 × 3 = 18). Each lane is color coded (darker color represents highly abundant proteins). Lanes A–M represent the regions of genome that correspond to highly abundant proteins

In total, 579 proteins were detected in the extracellular samples by LC–MS/MS, as shown in Additional file 2: Table S2A. The PCA plot (Additional file 1: Fig S1) and the correlation matrix (Additional file 1: Fig S2) reveal that the biological replicates clustered together and were highly correlated among replicates. Across sample conditions, protein abundance values obtained from simple substrates (glucose, xylose, and cellobiose) and complex substrates (Avicel, xylan, and switchgrass) grouped accordingly. As the focus was primarily on the extracellular proteins, intracellular proteins were computationally identified using Psort and Phobius and removed, leaving 192 proteins classified herein as extracellular (Additional file 2: Table S2B). Of these, 68 proteins contained predicted signal peptides. To further examine the genomic localization (and operon structures), the predicted and measured extracellular proteins were visualized on a circular genome atlas that depicts their abundance across the six substrates (Fig. 2). Biological replicates are shown in adjacent rings, and each substrate growth condition is indicated by a unique color. In general, there are 13 major regions (dark colored) along the chromosome (marked A–M in Fig. 2) that highlight sections of the genome that are translated into highly abundant proteins under all growth conditions. The most noteworthy cluster of highly abundant proteins belongs to region H, which encodes the major pectate lyases, the GDL family of glycosyl hydrolases (GH; GH-5, -9 and -48), and mannan *endo*-1,4-beta-mannosidases, as shown in Table 1. Many of the other regions identified consist of proteins with known functions linked to carbohydrate binding, uptake, and metabolism. However, regions C, D, L, and M contain highly abundant proteins with no annotated function. Of particular interest is region D, which consists of four proteins of unknown function (PUFs) localized together, perhaps indicative of co-regulation during lignocellulosic deconstruction/utilization.

**Table 1:**
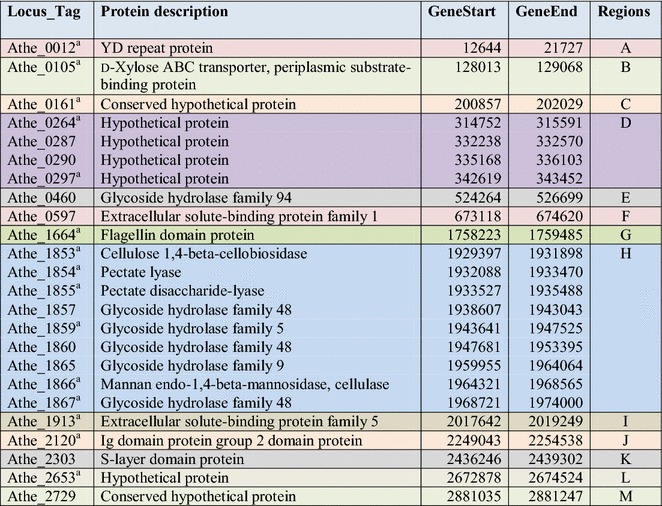
Most abundant extracellular proteome

^a^Core extracellular proteins

### The invariant core extracellular proteome

The core extracellular proteins that are abundant across all growth conditions but relatively invariant under any specific condition (i.e., *p* > 0.05 when compared across all substrates) were investigated as the first category. Among them, six proteins (Athe_1853, 1854, 1855, 1859, 1866, and 1867) are classified as CAZymes. Athe_1853, 1854, and 1855 are the pectate lyases A, B, and C, respectively, which cleave pectin, a major component of the primary cell walls of higher plants [48]. These proteins have been reported to be highly abundant in the secretomes of *C. bescii* and *C. obsidiansis* when grown on Avicel [10]. Previously, transcriptional analyses have reported these genes to be upregulated when *C. bescii* was grown on switchgrass [49] and downregulated on cellulose [25], both when compared to growth on glucose. Although gene expression was shown to be differential, the extracellular proteomic data presented here indicate that their abundances were fairly consistent across different substrates, and highly abundant even when cells are grown on simple monomeric substrates. This illustrates the complementary nature of transcriptomic and proteomic datasets, and demonstrates the need for integrated omics especially when studying extracellular environments where proteins/enzymes can accumulate, persist, and continue to function outside the observed induction of intracellular gene expression.

The other three core CAZymes identified, GHs Athe_1859, 1866, and 1867, are part of a multi-domain cellulase/hemicellulase gene cluster in the *C. bescii* genome known as the GDL [23, 25]. The GDL is known to be involved in processing of C5 and C6 sugars [17] and is located in region H of the genome atlas (Fig. 2 and Table 1), which includes other important GHs (Athe_1857, 1860, and 1865) that were differentially abundant across substrates (discussed below). In contrast, the GDL CAZymes Athe_1859, 1866, and 1867 were highly abundant and did not vary across substrates. Athe_1867 (CelA) is one of the most widely studied proteins in *C. bescii,* and was one of the most abundant extracellular proteins measured across all substrates. This megazyme is primarily involved in the deconstruction of crystalline cellulose, and consists of three carbohydrate-binding domains (3× CBM3) sandwiched between two catalytic domains, a processive endoglucanase (GH9) and an exoglucanase (GH48) [10, 20, 21, 50]. Similarly, Athe_1859 (consisting of GH5, two CBM3, and GH44—known as CbMan5A/Cel44a, a bifunctional mannanase) and Athe_1866 (CelB, which consists of GH5, three CBM3, and GH5) were also highly abundant. As noted above, Athe_1867 (CelA) and Athe_1859 are two of the three members responsible for almost complete deconstruction of microcrystalline cellulose (Avicel), which perhaps explains their persistent abundance in *C. bescii*’s secretome [23].

Other than the core CAZymes described above, several other proteins were found to be consistently abundant across all substrate classes. These include proteins involved in carbohydrate binding and transport (Athe_0012, 0105, 1913) or cell adhesion/potential carbohydrate recognition and motility (Athe_1664 and 2120). Of particular interest, however, were four proteins of unknown function (Athe_0161, 0264, 0297, and 2653) that were also highly abundant and independent of the carbohydrate substrate supplied. Their persistent abundance across all conditions suggests that they play a critical role in deconstruction or utilization of lignocellulosic material. In fact, Athe_0264 is one of the most abundant proteins in the entire extracellular proteome but its function has yet to be determined. There are no known orthologs of this protein, though the second best hit in BlastKOALA [51] matches to a multidrug efflux pump—an interesting connection but whose functional assignment would require additional experimental confirmation.

### Protein with varying abundance trends across growth substrates

One-way ANOVA generated a list of 115 extracellular proteins that were significantly (*p* value < 0.05) increased or decreased in abundance in at least one substrate condition. This list was further curated to highlight proteins that respond to a specific type of substrate or substrate class. The main comparisons included any substrate compared with all other substrates, C5 vs. C6 substrates, and simple vs. complex substrates. Glucose, cellobiose, and xylose were categorized as simple substrates, whereas Avicel, xylan, and switchgrass were categorized as complex substrates. Proteins that changed significantly in abundance (BH FDR adjusted *p* value < 0.05) in at least one comparison are shown in Additional file 3: Table S3. These differentially abundant proteins were then examined in more detail to ascertain their possible involvement in the deconstruction of specific substrates or substrate component.

**Fig. 3:**
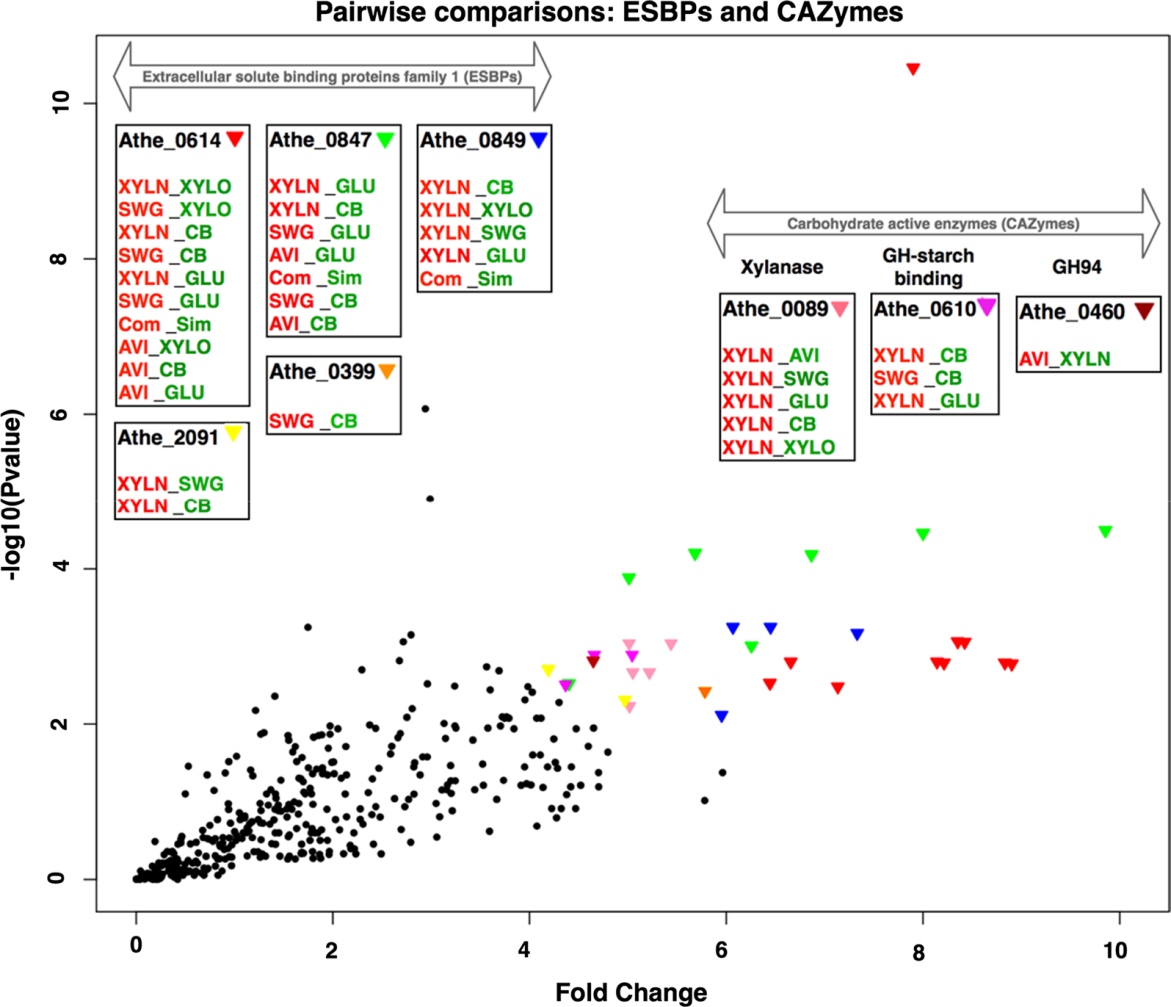
Scatterplot of absolute fold change (in log scale) and −log10 (*p* value) obtained by pairwise comparisons of ESBPs and CAZymes across all growth conditions. The most significant proteins (*p* value < 0.01 and fold change > ×4) are colored. Each point represents the *p* value and fold change obtained by the pairwise comparison. Each rectangular box represents the locus tag of protein along with the most significant comparisons. The red colored substrate means the high abundance of proteins and green color means low abundance of protein

Table 2 shows the overall distribution of protein class per substrate comparison. Overall, 43 differentially abundant proteins contained a signal peptide. Among 18 extracellular CAZymes measured, 11 were differentially abundant, with 7 containing CBMs (Table 3). Interestingly, CBM-containing CAZymes were most abundant on either switchgrass or xylan in the individual substrate pairwise comparisons. ESBPs are another broad category of extracellular proteins that have non-catalytic extracellular activities [52]. Out of 22 predicted ESBPs, 14 were differentially abundant. As both CAZymes and ESBPs are important to the process of lignocellulose deconstruction and utilization, and are known to be extracellular, both protein classes were visualized as a scatter plot (Fig. 3) based on their absolute fold changes and *p* values (Table 3) obtained through pairwise comparison. Clearly, enzymes suspected to be involved in the deconstruction of switchgrass and xylan are the most differentially abundant, as shown in Additional file 3: Table S3 and Fig. 3. ESBPs (Athe_0847, 0849, 0614) were categorically more abundant in complex substrates like xylan, switchgrass, and Avicel when compared to simple substrates, with Athe_0849 more specific to the C5 polymer xylan relative to SWG. In addition to these ESBPs, Athe_0089 was highly specific to xylan. This is expected, as Athe_0089 is functionally categorized as an *endo*-1,4-beta-xylanase and thus would be an important player in the deconstruction of pure xylan. Overall, the most significant ESBPs and CAZymes (color coded in Fig. 3) revealed higher abundance in xylan, switchgrass, and/or Avicel when compared to other simple substrates, and thus strongly implicate their importance to the deconstruction and utilization of complex substrates by *C. bescii*.

**Table 2:** Extracellular proteome summary

	All vs. all	Simple vs. complex	C5 vs. C6	Union
SignalP	33	13	25	43
CAZymes	8	6	3	11^a^
ESBPs	12	9	5	14
PUFs	25	13	21	37

^a^Out of 11 CAZymes, 7 consist of CBMs

**Table 3:** Differentially abundant CAZYmes in different growth conditions

LocusTag_ProteinDescription	Comparison (subA_subB)^a^	*p* value	subA–subB (fold change^a^)	CBM
Athe_0610_glycoside hydrolase starch-binding	CB_SWG	0.00	− 4.66	Yes
Athe_0610_glycoside hydrolase starch-binding	GLU_SWG	0.00	− 3.98	Yes
Athe_0610_glycoside hydrolase starch-binding	GLU_XYLN	0.00	− 4.37	Yes
Athe_0610_glycoside hydrolase starch-binding	Simple_Complex	0.01	− 2.81	Yes
Athe_0610_glycoside hydrolase starch-binding	CB_XYLN	0.00	− 5.04	Yes
Athe_0610_glycoside hydrolase starch-binding	C6_C5	0.01	− 2.69	Yes
Athe_0460_glycoside hydrolase 94	GLU_CB	0.02	− 1.55	No
Athe_0460_glycoside hydrolase 94	XYLN_XYLO	0.00	− 2.68	No
Athe_0460_glycoside hydrolase 94	GLU_XYLN	0.00	1.41	No
Athe_0460_glycoside hydrolase 94	GLU_XYLO	0.01	− 1.27	No
Athe_0460_glycoside hydrolase 94	AVI_SWG	0.02	4.8	No
Athe_0460_glycoside hydrolase 94	AVI_XYLN	0.00	4.65	No
Athe_0460_glycoside hydrolase 94	AVI_XYLO	0.01	1.97	No
Athe_0460_glycoside hydrolase 94	AVI_CB	0.03	1.69	No
Athe_0460_glycoside hydrolase 94	CB_XYLN	0.00	2.96	No
Athe_0460_glycoside hydrolase 94	GLU_AVI	0.00	− 3.24	No
Athe_0459_glycoside hydrolase 94	AVI_SWG	0.04	4.7	No
Athe_0459_glycoside hydrolase 94	AVI_XYLN	0.04	5.96	No
Athe_0459_glycoside hydrolase 94	AVI_CB	0.01	1.54	No
Athe_0459_glycoside hydrolase 94	GLU_AVI	0.03	− 2.91	No
Athe_2028_glycoside hydrolase family 4	Simple_Complex	0.04	1.16	No
Athe_1857_glycoside hydrolase family 48	AVI_XYLN	0.03	− 1.63	Yes
Athe_1857_glycoside hydrolase family 48	AVI_XYLO	0.02	− 2.13	Yes
Athe_1857_glycoside hydrolase family 48	AVI_CB	0.03	− 2.96	Yes
Athe_1857_glycoside hydrolase family 48	Simple_Complex	0.01	1.21	Yes
Athe_1857_glycoside hydrolase family 48	GLU_AVI	0.04	1.9	Yes
Athe_0089_Endo-1,4-beta-xylanase	XYLN_XYLO	0.00	5.01	Yes
Athe_0089_Endo-1,4-beta-xylanase	GLU_XYLN	0.00	− 5.05	Yes
Athe_0089_Endo-1,4-beta-xylanase	XYLN_SWG	0.00	5.22	Yes
Athe_0089_Endo-1,4-beta-xylanase	AVI_XYLN	0.00	− 5.44	Yes
Athe_0089_Endo-1,4-beta-xylanase	CB_XYLN	0.01	− 5.02	Yes
Athe_0089_Endo-1,4-beta-xylanase	C6_C5	0.02	− 2.66	Yes
Athe_1860_glycoside hydrolase family 48	XYLN_XYLO	0.03	− 0.53	Yes
Athe_1860_glycoside hydrolase family 48	AVI_SWG	0.05	− 2.14	Yes
Athe_1860_glycoside hydrolase family 48	XYLN_SWG	0.05	− 0.72	Yes
Athe_1860_glycoside hydrolase family 48	AVI_XYLO	0.05	− 1.95	Yes
Athe_1860_glycoside hydrolase family 48	AVI_CB	0.05	− 2.89	Yes
Athe_1860_glycoside hydrolase family 48	Simple_Complex	0.03	1.02	Yes
Athe_0609_pullulanase, type I	Simple_Complex	0.00	− 2.29	Yes
Athe_0609_pullulanase, type I	C6_C5	0.03	− 1.99	Yes
Athe_0152_Acetyl xylan esterase	GLU_CB	0.03	4.11	No
Athe_0152_Acetyl xylan esterase	GLU_XYLN	0.03	4.03	No
Athe_0594_Cellulase	Simple_Complex	0.01	− 2.05	Yes
Athe_1865_glycoside hydrolase family 9	AVI_SWG	0.04	− 1.75	Yes
Athe_1865_glycoside hydrolase family 9	AVI_XYLN	0.01	− 1.8	Yes
Athe_1865_glycoside hydrolase family 9	AVI_XYLO	0.01	− 1.81	Yes
Athe_1865_glycoside hydrolase family 9	AVI_CB	0.04	− 1.89	Yes
Athe_1865_glycoside hydrolase family 9	C6_C5	0.04	− 0.91	Yes

^a^The fold changes are in log scale. If the fold change is positive (+), the protein is significantly more abundant in substrate (left side) when compared substrate (right side) in the comparison column. Similarly, if the fold change is negative (−), the protein is significantly more abundant in substrate (right side) when compared substrate (left side) in the comparison column

**Fig. 4:**
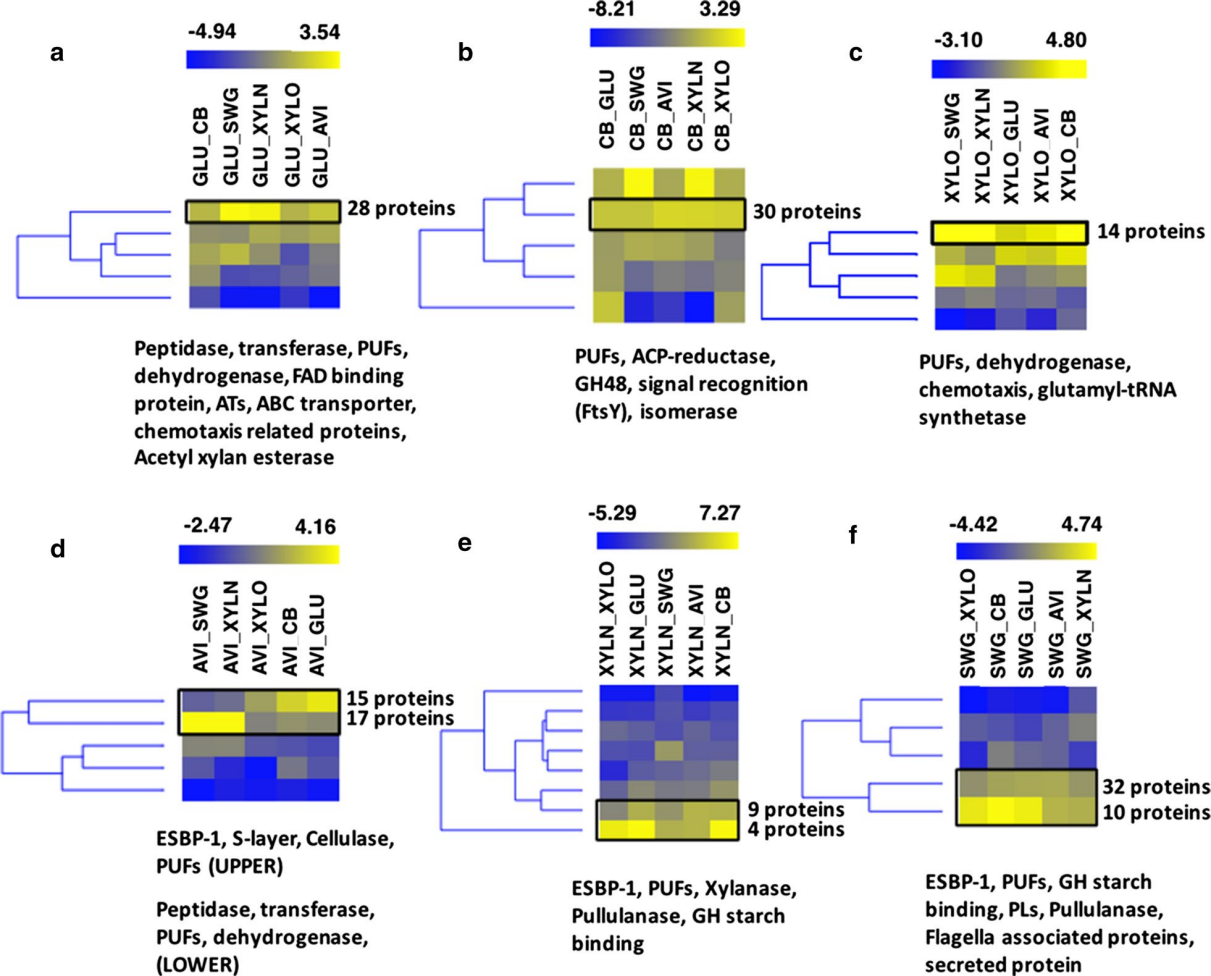
Clustering of proteins in a heat-map based on fold-change difference when one substrate is compared versus all five other substrates. **a** Glucose; **b** cellobiose; **c** xylose; **d** Avicel; **e** xylan; and **f** switchgrass. The darker yellow color in the heat-map refers to a group of proteins having maximum fold change and a darker yellow color is progressively decreasing in fold-change difference when a substrate is compared with another substrate. The black rectangular box refers to the protein clusters that have highest fold-change difference versus another substrate. The functional description of the proteins in these clusters are mentioned underneath each heat-map

The suite of extracellular enzymes appears to be highly dependent on the nature and complexity of the growth substrate. To further explore substrate-dependent protein abundance, the differentially abundant proteins identified by ANOVA above were first segregated by substrate, and then individual proteins clustered according to their fold-change values when compared to all other substrates (Fig. 4 and Additional file 4: Table S4). Growth on the monosaccharides glucose and xylose show increased abundance of proteins involved in signal transduction, chemotaxis, ABC transport, and other broad functional categories (Fig. 4a, c). With regard to the disaccharide cellobiose (Fig. 4b), several PUFs as well as GH48-related proteins were detected in higher abundance when compared to other substrates. Growth on the complex, C6 polysaccharide Avicel revealed two kinds of trending proteins clusters that exhibited contrasting abundance patterns (Fig. 4d). One cluster contains proteins that were abundant when compared to simple sugars, but less abundant when compared to xylan and switchgrass. Since both switchgrass and xylan contain C5 polymers, it is possible that this cluster contains proteins specific to C5 deconstruction or utilization. This cluster mainly consists of ESBP-1, PUFs, and S-layer domain proteins. The other cluster in Fig. 4d comprises proteins that were highly abundant when compared to switchgrass and xylan, but lower when compared to simple sugars—the majority of which were identified and discussed earlier in glucose and xylose.

A pair of CAZymes (Athe_0459, 0460) were significantly more abundant in Avicel compared to other growth conditions. These proteins possess cellobiose/cellodextrin phosphorylase activities, and were also found to be significantly more abundant in cellobiose as compared to glucose. Interestingly, their abundances were higher in Avicel (2×) compared to cellobiose alone, the latter of which being the obvious substrate target. Since the sample collection occurred during mid-log phase, other cellulase enzymes likely have already begun solubilizing and deconstructing the cellulose (Avicel) into cellobiose, thus increasing the abundances of these enzymes. These were previously annotated as glycosyltransferase 36, and have been updated to GH94 in the CAZY database [53]. For comparison, a study in the related cellulolytic bacterium, *Clostridium cellulolyticum* ATCC 35319, reported that cellobiose/cellodextrin phosphorylase genes (GH94: Ccel_3412 and 2109) were expressed when the organism was grown on cellulose (Avicel PH101) [54]. Although GH48 family CAZymes (Athe_1860, Athe_1857:CelF) were detected as abundant enzymes in all conditions, Athe_1860 (consisting of GH74, GH48, and two CBM3) was significantly more abundant in cellobiose as compared to Avicel, and was also more abundant in xylose and switchgrass as compared to xylan and Avicel. In contrast, a previous study detected Athe_1860 as less abundant when grown in crystalline cellulose [10]. Similarly, CelF, a xylanase/cellobiohydrolase, was significantly more abundant in xylan, xylose, cellobiose, and glucose as compared to Avicel.

As opposed to the other substrates analyzed, switchgrass and xylan (Fig. 4e, f) are both composed of C5 polysaccharides and correlated with increased abundance of ESBP family 1 proteins, which play major roles in the transport/uptake of deconstructed oligomers using ATP [55]. Pullulanase, an amylolytic debranching enzyme, was highly abundant during growth under both conditions, and even more so during growth on xylan compared to switchgrass. This is reasonable given its debranching activity [56], which is discussed below. This is also in line with observations for pectate lyase, which was also highly abundant in these C5-containing biopolymers and known to be involved in the deconstruction of plant cell walls [57]. Finally, several flagellar associated proteins were highly abundant during growth on switchgrass and xylan and are likely associated with either motility behavior or adhesion to these specific substrates.

### Highly abundant proteins involved in C5 substrate utilization

**Fig. 5:**
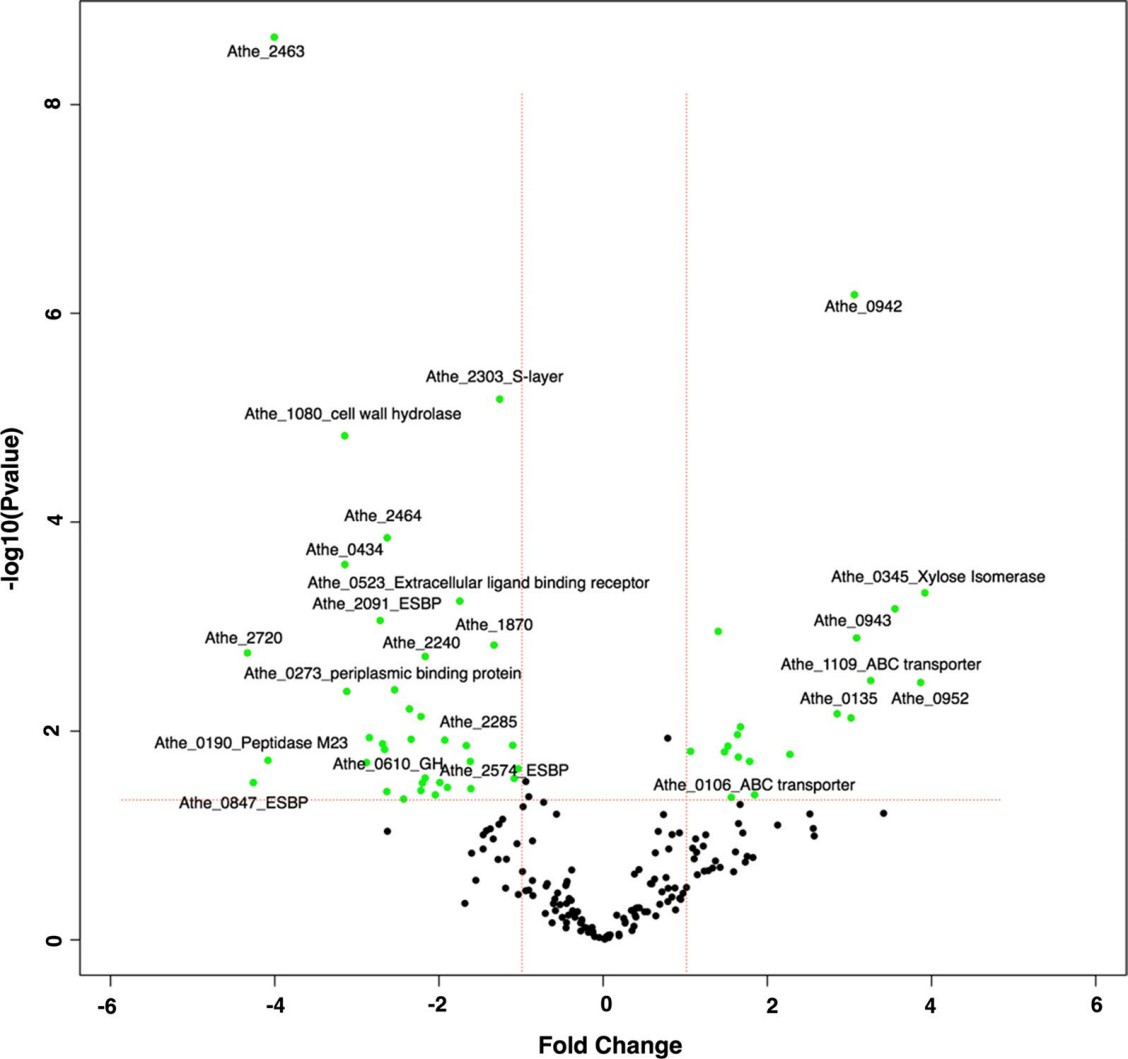
Volcano plot showing differentially abundant proteins based on fold change versus *t* test probability. The plot is obtained with the proteomic approach when comparing extracellular proteome metrics obtained by growing *C. bescii* in C5 substrates versus C6 substrates. Green dots represent the proteins that have a *p* value < 0.05 and >  2 fold change. The green dots on the left side of the plot are the proteins that are differentially more abundant and specific in C5 substrates. Similarly, green dots on the right side of the plot are the proteins that are differentially more abundant and specific in C6

Several differentially abundant enzymes were detected when comparing C5 vs. C6 substrates. Switchgrass was not included in this analysis because of its heterogeneous composition (both cellulose and hemicellulose). In total, 59 proteins were differentially abundant, with 39 proteins specific to growth on C5 and the remaining 20 proteins specific to C6 (Additional file 3: Table S3). The volcano plot in Fig. 5 illustrates the proteins that were C5-specific (left side) or C6-specific (right side). Four ESBPs (Athe_0523, 2091, 2574, 0847) were significantly more abundant on C5 substrates relative to C6. In fact, not a single extracellular binding protein was more abundant when *C. bescii* was growth on C6 substrates. This indicates that the extracellular binding proteins may be more specific towards hemicellulose deconstruction—perhaps a more natural “condition,” especially in the case of *C. bescii* growing in the wild. This was also observed among the 14 PUFs that were specific to C5 substrates (Fig. 5 and Additional file 3: Table S3). Cell wall hydrolase (Athe_1080) was more abundant on C5 substrates as well, with a fold-change difference of more than 8×. The GO-based functional annotation of this enzyme revealed that it is involved in peptidoglycan catabolism, specifically hydrolyzing the link between *N*-acetylmuramoyl residues and l-amino acid residues in cell wall glycopeptides [58].

It therefore appears that proteins capable of extracellular solute binding and hydrolase activity were more specific towards C5 relative to C6 substrates. This trend holds true for other proteins linked to hemicellulose deconstruction, such as Athe_2091 and 0523. Athe_2091 was significantly more abundant during growth on xylan than glucose, cellobiose, Avicel, and switchgrass. Likewise, Athe_0523 was more abundant (by ~ 4×) compared to other substrates during growth on xylose and xylan, and was significantly more abundant on xylose compared with switchgrass and Avicel. Evaluation by gene ontology (GO) terms revealed that these C5-specific proteins likely hydrolyze *O*-glycosyl compounds specifically.

In contrast to C5-specific proteins, ten proteins were more abundant under growth with C6 substrates. Xylose isomerase domain protein (Athe_0345) was ~ 16× more abundant in C6 relative to C5 (with a very low *p* value). Even though the name suggests xylose isomerase activity, a BLAST search of Athe_0345 revealed 10 hits of 93–97% similarity with sugar phosphate isomerase, suggesting that this protein might not be specific to xylose but instead have a more general function towards C6 substrates. The results of BLAST hits and CDD domain matches are shown in Additional file 1: Fig. S3. In addition to Athe_0345, a couple of ABC transporter-related proteins (Athe_1109 and 0106) were more abundant in C6 substrates, likely indicating the importance of transport of glucose molecules. Interestingly, three proteins of unknown function were specific to C6 and were highly abundant, suggesting their potential importance to C6 utilization.

Although switchgrass was excluded from the basic comparison discussed above because it contains both C5 and C6 sugars, it was still important to analyze this substrate in the context of all others and was thus included in the hierarchical clustering of abundance trends shown in Additional file 1: Fig. S4. As expected, the extracellular protein profiles measured for xylan and xylose (C5) clustered together but were divergent from Avicel, cellobiose, and glucose (C6), which grouped similarly. Switchgrass was notably sandwiched between C5 and C6 substrates, with some proteins sharing abundance patterns similar to those observed for C5 sugars and others to C6. The highly abundant proteins measured in switchgrass that previously showed C5- or C6-only abundance patterns (i.e., sans switchgrass) are perhaps critical to the deconstruction of those specific substrates. C5-specific proteins that were highly abundant during growth on switchgrass also are highlighted in pink (Additional file 1: Fig. S2). Briefly, key GHs (Athe_1865, 0610, 0609), cell wall hydrolase (Athe_1080), ESBPs (Athe_2574, 0847), periplasmic binding proteins (Athe_0273), and PUFs (Athe_2464, 2720, 2719, 2368) were C5 specific proteins that were highly abundant in switchgrass as well. These proteins likely drive the solubilization and utilization of C5 polymers derived from switchgrass.

### ESBPs and GHs are critical for deconstruction of complex substrates

**Fig. 6:**
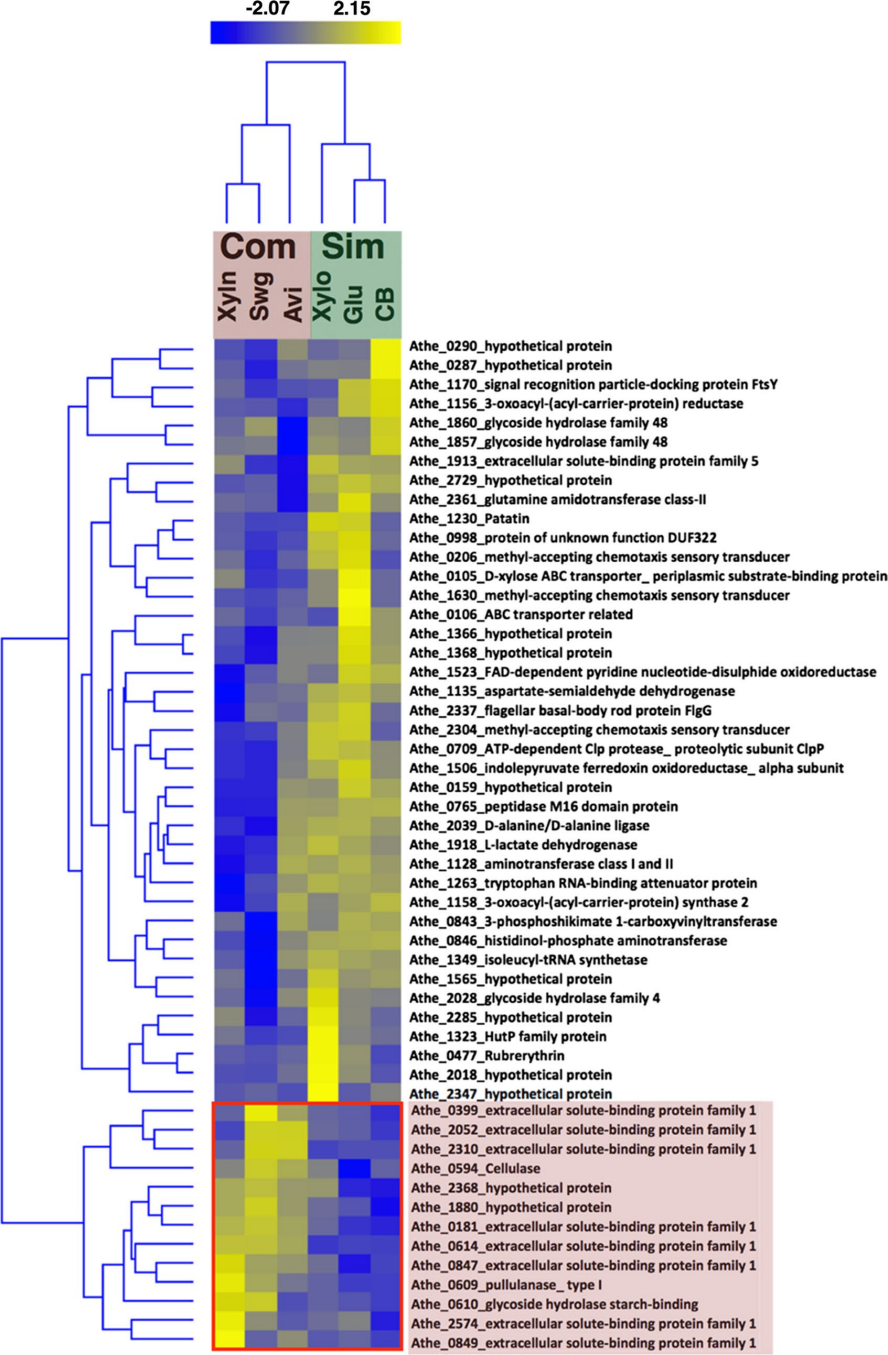
Hierarchical clustering based on individual protein *z*-score of the significant proteins (*p* value < 0.05) obtained from pairwise comparison of simple substrates versus complex substrates comparison. The red rectangular box shows the proteins that were most differentially abundant and specific to complex substrates. The reported abundances are *z*-scores of log2 transformed intensity

The next goal was to investigate the effect of *general* substrate complexity on the extracellular enzyme inventory. Thus, a comparison was made between simple and complex growth substrates; glucose, cellobiose, and xylose were categorized as simple substrates, and Avicel, xylan, and switchgrass were categorized as complex substrates. A total of 53 proteins were differentially abundant (*p* value < 0.05) between simple and complex growth conditions. A total of 40 proteins had increased abundances in simple substrates compared to only 13 proteins in complex substrates Fig. 6. Out of the 40 proteins more abundant on simple substrates, only three CAZYmes were identified. Athe_1857 (CelF; a xylanase/cellobiohydrolase, which consists of GH10, 2 CBMs, and GH48 for deconstruction of polymers) was slightly more abundant on simple substrates, with most of the increased abundance driven by *C. bescii* growth on cellobiose. Similarly, Athe_1860 (a probable cellobiohydrolase consisting of GH74, 2 CBMs, and GH48) and Athe_2028 (a glucosidase and galactosidase involving NAD^+^ consisting of a GH4 domain) were also specific to growth on simple substrates.

In contrast, among 13 proteins specific for growth on complex substrates (indicated by red box in heat-map, Fig. 6), 8 were ESBP family 1 (Athe_0614, 0181, 0847, 2310, 0849, 2052, 0399, and 2574), 3 were CAZymes: pullulanase, type I (Athe_0609), cellulase (Athe_0594—CelD), glycoside hydrolase starch-binding (Athe_0610), and 2 were proteins of unknown function (Athe_1880, 2368). Three of the most abundant proteins specific to complex substrates (by ~ 8× to 256×) are Athe_0614, 0181 and 0847, which are ESBP family proteins that are functionally relevant to ABC transport. Since they were the most differentially abundant ESBPs, they likely have crucial roles in the deconstruction/utilization of complex substrates. Further differentiation among the ESBPs identified in the complex substrate cluster was observed. For example, Athe_0399, 2310, and 2052 were more abundant only when *C. bescii* was grown on switchgrass or pure cellulose. This indicates that these ESBPs may be specifically involved in C6 oligomer deconstruction and uptake, since switchgrass and Avicel both contain C6 polymers. Conversely, ESBPs Athe_0849 and 2574 appear to share a proclivity towards C5 sugars present in xylan and, to a lesser extent, switchgrass—an observation that is especially evident with Athe_2574.

ESBPs are one large group of proteins that remains understudied in *C. bescii*. These proteins are also termed as non-catalytic plant cell wall binding proteins (PWBP). Although they are known to bind a wide range of substrates, they have the highest affinities for plant cell wall xylan and pectin [52]. In this study, all the significant ESBPs were highly abundant in switchgrass and/or xylan. Since switchgrass and xylan both contain C5 polymers, the roles of ESBPs are likely crucial for the deconstruction and utilization of these more complex substrates. While the KEGG database indicates that these proteins are ABC transporter related, further inquiry with InterProScan identified potentially novel functional domains present in these proteins. For example, a Leu/Ile/Val-binding protein family signature was detected in Athe_1388 and 0523, suggesting that these proteins play a role in the transport of branched-chain amino acids. Athe_2574 was predicted to have a maltose binding protein signature, suggesting a possible role of binding and transport of linear oligosaccharides. Athe_0614 was the most abundant ESBP in complex substrates compared to simple substrates. This protein has 34% identity and 89% similarity to the xylooligosaccharide binding protein (XBP1) from *Caldanaerobius polysaccharolyticus* and its function in xylan utilization has been previously reported [13]. Accordingly, our study found this protein to be the most highly abundant during growth on xylan and switchgrass.

Besides the family 1-type ESBPs, 5 additional proteins are specifically more abundant in complex substrates relative to simple substrates. These include GHs Athe_0594, 0609, and 0610 and PUFs Athe_1880 and 2368. Athe_0609, a pullulanase type 1 enzyme, seems to be critical for the deconstruction of complex substrates. A search with InterProScan revealed its domain structure to contain an alpha-amylase domain (GH13), CBM48, CBM20, starch binding domain, and pullulanase type I domain. Pullulanase is a debranching enzyme involved in the hydrolysis of (1→6)-α-d-glucosidic linkages in pullulan, amylopectin, and glycogen, as well as in the α- and β-limit dextrins of amylopectin and glycogen [56, 59]. The major molecular functions of Athe_0609 are hydrolases activity and carbohydrate binding. Like its genomic neighbor, Athe_0610, consisting of CBM20 and a starch binding domain, was significantly more abundant on switchgrass and xylan relative to cellobiose and glucose. Since switchgrass also contains xylan, Athe_0610 might act specifically on xylan (C5) polymers. Both of these enzymes are encoded in the same operon and possess CBM20 and starch binding domains, suggesting their roles may be more diverse than their annotations suggest. Athe_0594, otherwise known as CelD (note that a close homolog to this, Csac_0698, was previously characterized [60]), an *endo*-1,4-glucanase consisting of GH5 and CBM28, was also specific to growth on complex substrates. Known activities for GH5 domains include *endo*-β-1,4-glucanase; *endo*-β-1,4-xylanase; β-glucosidase; β-mannosidase; glucan β-1,3-glucosidase; *exo*-β-1,4-glucanase/cellodextrinase; cellulose β-1,4-cellobiosidase—all of which are vital for deconstruction of complex substrates.

### Proteins of unknown function

**Fig. 7:**
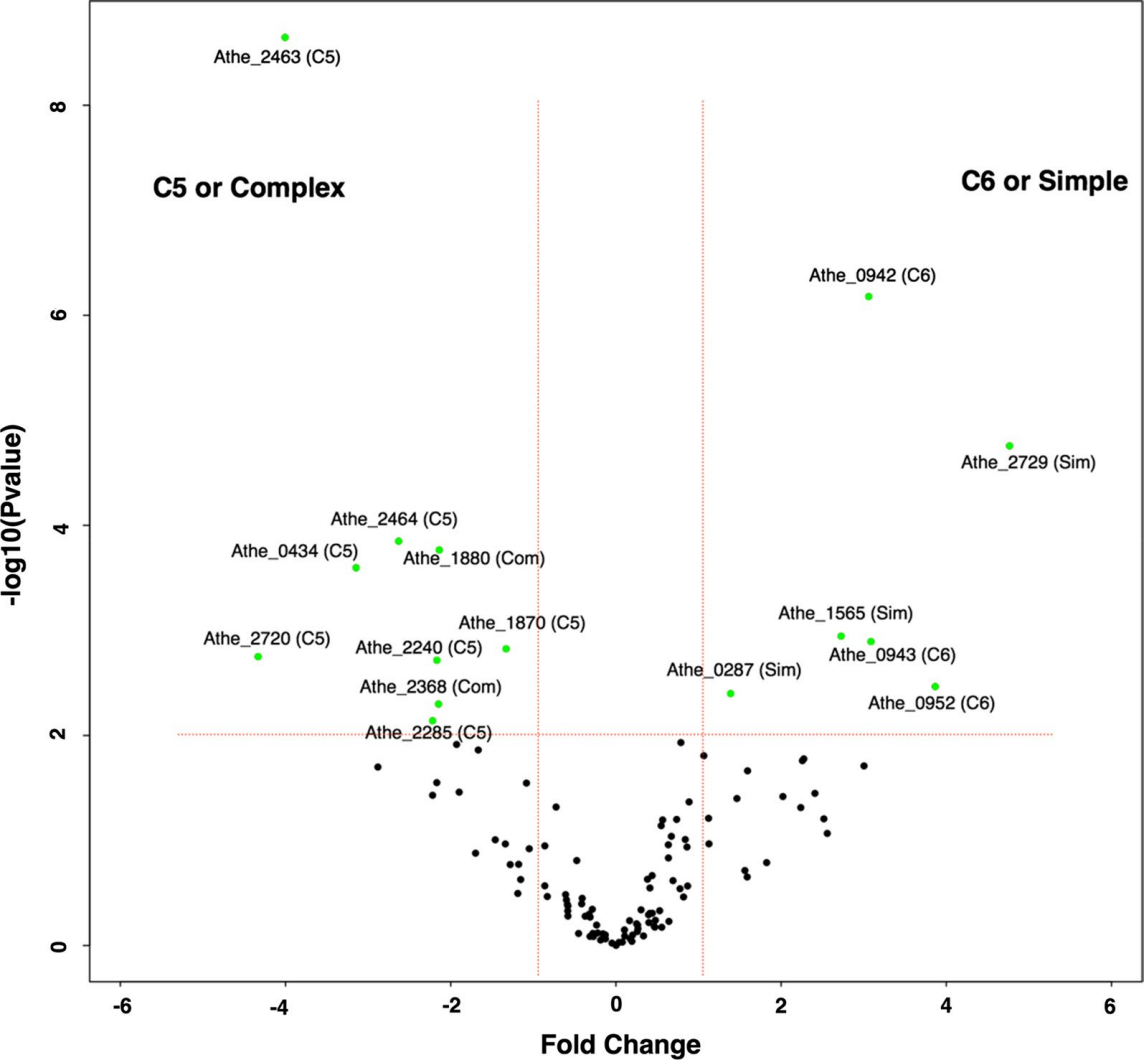
Volcano plot showing differentially abundant proteins of unknown functions. The plot is a merged volcano plot obtained with the proteomic approach when comparing extracellular proteins of unknown functions obtained by growing *C. bescii* in C5 substrates versus C6 substrates and complex substrates versus simple substrates. Green dots represent the proteins that have a *p* value < 0.01 and > ×2 fold change. The green dots on the left side of the plot are the proteins that are differentially more abundant in C5/Complex substrates (as mentioned in the parentheses). Similarly, green dots on the right side of the plot are the proteins that are differentially more abundant in C6/Simple substrates (as mentioned in the parentheses)

Function is often difficult to ascertain based on bioinformatic annotation alone. Thus, protein abundance patterns observed across different growth substrates or substrate classes may lend evidence to their generalized function, if even at a high level, i.e., C5-responsive extracellular protein. Thus, the meta information gleaned from analyses like these should be useful for uncovering the function of substrate responsive PUFs. To help direct future functional genomic efforts involving extremely thermophilic, cellulolytic organisms such as *C. bescii* during its deconstruction and utilization of complex biomass substrates, information on all PUFs that were measured in this analysis with their relative abundances across substrates is provided (Additional file 5: Table S5). Furthermore, InterProScan was employed to provide as much up-to-date information as possible regarding the functional annotation of measured PUFs (Additional file 6: Table S6). InterProScan revealed some PUFs that could be functionally re-categorized. For example, Athe_2347 contains Pfam domain PF12679, which functions as an ABC-2 family transporter. This protein was detected primarily during growth on xylose but was also significantly more abundant on all of the simple growth substrates. Similarly, Athe_2368, which contains Pfam domain PF09822 that functions as an ABC-type uncharacterized transporter, was significantly more abundant during growth on both complex and C5 substrates. Athe_1880, specific towards complex substrates (Figs. 6, 7), contains Pfam domain PF13544, suggesting it is a surface proteins and possibly part of type IV pilin N-term methylation site GFxxxE and involved in secretion broad ranges of protein substrates [61].

Across all samples, a total of 100 proteins of unknown function (PUF) were detected, with 58 categorized as potential extracellular proteins. Thirty-seven of these extracellular PUFs exhibited significant differences in abundance across the different growth conditions, as shown in Additional file 5: Table S5. Among these 37 proteins, only 12 contained recognizable signal peptides. When only signal peptide-containing proteins were examined, Athe_2463 was the most differentially abundant (by almost ~ 16×), showing high abundance on xylan and xylose compared to all other substrates, and suggesting a potential role in C5-specific extracellular activity (Fig. 7, Additional file 1: Fig. S2). Similarly, Athe_2464 was highly abundant on xylan, xylose, and switchgrass relative to other substrates. Since both PUFs are encoded in the same operon, their co-expression during growth on C5 substrates perhaps underscores their importance in lignocellulose deconstruction and suggests potential high-value targets for future functional characterization efforts. Athe_0434 is another xylan-specific protein that showed significantly more abundance in xylan-grown cells as compared to those grown on xylose, glucose, switchgrass, and Avicel. In fact, Athe_2463, 2464, 0434, 2720, 1870, and 1397 were all significantly more abundant on C5 substrates as compared to C6, as shown in the volcano plot (Fig. 7). Athe_1870, 1871 (*p* value = 0.013) are localized in the genomic region very close to GDL, and thus are likely related to glycoside hydrolase function specific to C5 substrates. In contrast, Athe_0942 and 0943 were significantly more abundant on C6 substrates compared to C5 substrates, by almost ~ 16× to 32× and thus could play a significant role in the C6 utilization metabolism.

In total, this work reports one of the most detailed characterizations of PUFs for a microbial secretome. In most studies, PUFs are largely ignored, since it is difficult to extract much meaningful information from them. The key for this study is the ability to link them to specific growth conditions, and thereby begin to tease out functional aspects which might lead to more definitive identifications/annotations. When combined with enhanced bioinformatic methods for examining potential domain structures, this type of approach might open a new window to exploring this previously discarded molecular family.

## Conclusions

This study was designed to examine changes in the extracellular protein inventory of *C. bescii* when the cellulolytic organism was grown on a variety of bioenergy-relevant substrates in order to identify key proteins responsible for substrate-specific deconstruction and/or utilization. The substrates chosen ranged from simple (monomeric) to complex (polymeric) and varied in their general composition (C5 or C6 sugars). Lignocellulosic biomass (switchgrass) was included to provide not only a real-world deconstruction scenario but also to ‘bridge the gap’ between specific C5 and C6 model substrates. The results showed that the nature of the carbon substrate (i.e., C5 vs. C6) and complexity of the lignocellulose drive the abundance patterns of most of the extracellular solute binding proteins (ESBPs), glycoside hydrolases (GHs), and proteins of unknown functions (PUFs). This work found that most of the proteins encoded by glucan degradation locus (GDL) was consistently highly abundant on all growth conditions and thus constitute much of the core extracellular proteome. However, other GHs (Athe_0609, 0610) are strongly linked to the deconstruction and utilization of C5/complex substrates. Along with these GHs, certain ESBPs (Athe_0614, 2368) appear to be vital for xylan utilization and ABC transport. Interestingly, some PUFs (Athe_2463, 2464) were strongly C5 substrate specific, highlighting their possible roles as potential xylanases. Thus, this study not only provided detailed information about the diversity and substrate specificity of enzymes, but also provided the research community with potential genomic targets for metabolic engineering of potentially desirable phenotype growth characteristics.

## Additional files

**Additional file 1.** Additional table and figures.

**Additional file 2: Table S2.** Normalized matched ion intensity measurement using mass spectrometry.

**Additional file 3: Table S3.** Significantly differentially abundant protein in pairiwise comparisons.

**Additional file 4: Table S4.** Substrate level fold-change matrixes of significant proteins (*p* value < 0.05 ANOVA).

**Additional file 5: Table S5.** Significantly differentially abundant Proteins of Unknown Functions (PUFs) obtained by binary substrates comparison.

**Additional file 6: Table S6.** Interproscan annotations of significant Proteins of Unknown Functions (PUFs) that contains signal peptide and obtained by binary substrates.
