# Supplementary material for: The diversity and specificity of the extracellular proteome in the cellulolytic bacterium Caldicellulosiruptor bescii is driven by the nature of the cellulosic growth substrate
Source: Biotechnol Biofuels. 2018 Mar 23;11:80. doi: 10.1186/s13068-018-1076-1 (PMC5865380; doi:10.1186/s13068-018-1076-1)
Supplement: Supplementary file 1 — Additional file 1. Additional table and figures. [file 13068_2018_1076_MOESM1_ESM.docx]

Additional Materials for

**The diversity and specificity of the extracellular proteome in the cellulolytic bacterium *Caldicellulosiruptor bescii* is driven by the nature of the cellulosic growth substrate**

Suresh Poudel^1,3,4^; Richard J. Giannone^2,3^; Mirko Basen^5,6^; Intawat Nookaew^1,3,8^; Farris L. Poole, II^3,5^; Robert M. Kelly^3,7^; Michael W.W. Adams^3,5^; and Robert L. Hettich^2,3^*

^1^Biosciences Division; ^2^Chemical Sciences Division; ^3^BioEnergy Science Center at Oak Ridge National Laboratory, Oak Ridge, TN 37831, USA ^4^Department of Genome Science and Technology, University of Tennessee, Knoxville, TN 37996, ^5^Department of Biochemistry and Molecular Biology, University of Georgia, Athens, GA 30602, ^6^current address: Department of Molecular Microbiology and Bioenergetics, Institute of Molecular Biosciences, Johann Wolfgang Goethe University, Frankfurt Am Main, Germany, ^7^ Department of Chemical and Biomolecular Engineering, North Carolina State University, Raleigh, NC 27695, ^8^current address: Department of Biomedical Informatics, College of Medicine, University of Arkansas for Medical sciences, Little Rock, AR, USA, 72205

**Table S1. Growth parameters, substrate consumption, and product formation of *C. bescii* on different substrates.** *C. bescii* cultures were grown at 78°C in complex medium with 5 g L^–1^ of different substrates. Cell densities and metabolite concentrations were determined prior to harvesting the cells for proteome analysis in the exponential growth phase (n=3; ±SD; *n.d.* not determined).

|  | **Glucose** | | **Cellobiose** | | **Crystalline cellulose** | | **Xylose** | **Xylan** | **Unpretreated switchgrass** | | |
| --- | --- | --- | --- | --- | --- | --- | --- | --- | --- | --- | --- |
| **Cell density (cells mL^–1^)** | | **9.5E+07** *±2.5E+07* | | **1.5E+08** *±1.3E+07* | | **1.0E+08** *±1.7E+07* | **8.0E+07***±1.1E+07* | **9.5E+07** *±2.7E+07* | | **1.0E+08** *±1.3E+07* |  |
| **Growth rate (h^-1^)** | | **0.67** | | **0.57** | | **0.37** | **0.24** | **0.43** | | **0.34** |  |
| **Acetate (mM)** | | **2.2** *±0.4* | | **1.7***±0.2* | | **2.3***±0.0* | **3.1***±0.6* | **2.6***±0.2* | | **1.7***±0.2* |  |
| **Lactate (mM)** | | **0.0***±0.0* | | **0.1***±0.2* | | **2.6***±0.3* | **0.9***±0.4* | **0.3***±0.2* | | **0.0***±0.0* |  |
| **Substrate consumed (mM)** | | **2.1***±0.5* | | **0.8***±0.0* | | **n.d.** | **5.0***±1.5* | **n.d.** | | **n.d.** |  |

Fig. S1 . **Correlation plot of the Pearson correlation values when different conditions were compared within biological replicates and between samples.**

Fig. S2. **PCA plot illustrating how well the biological replicates grouped together, and the clear distinction between the growth conditions**


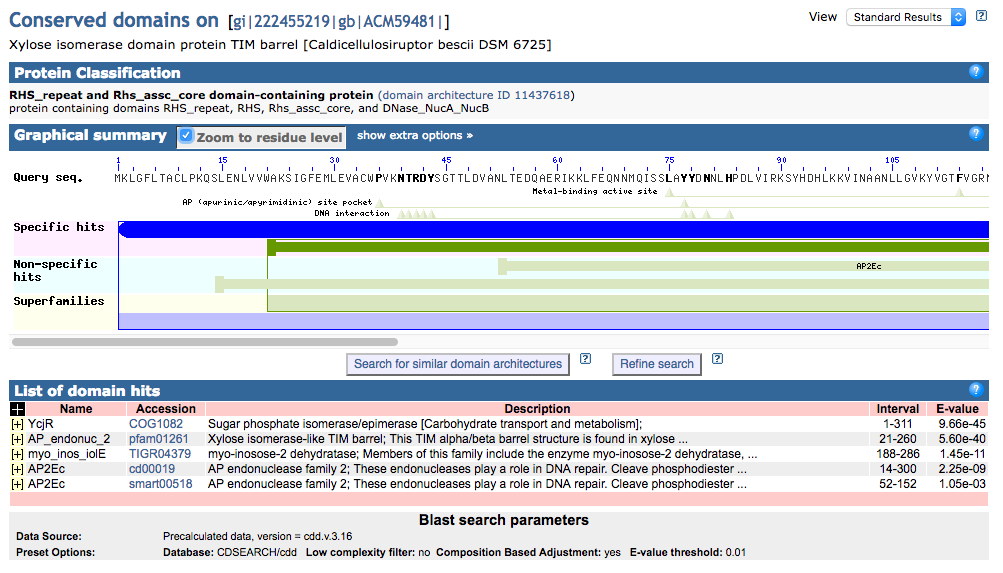


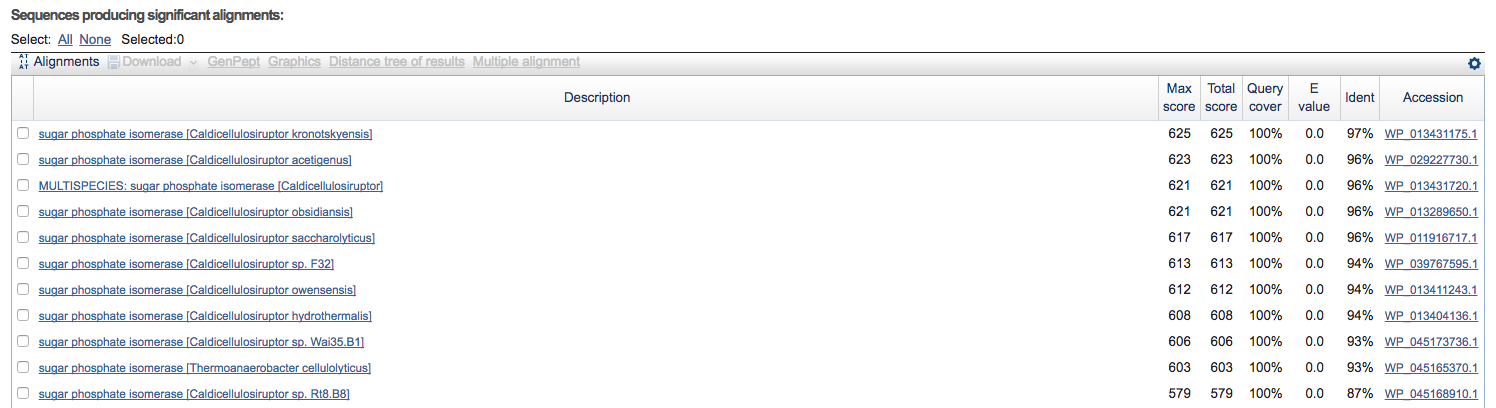


**Fig. S3. Conserved domain results for xylose isomerase activity of Athe_0345**


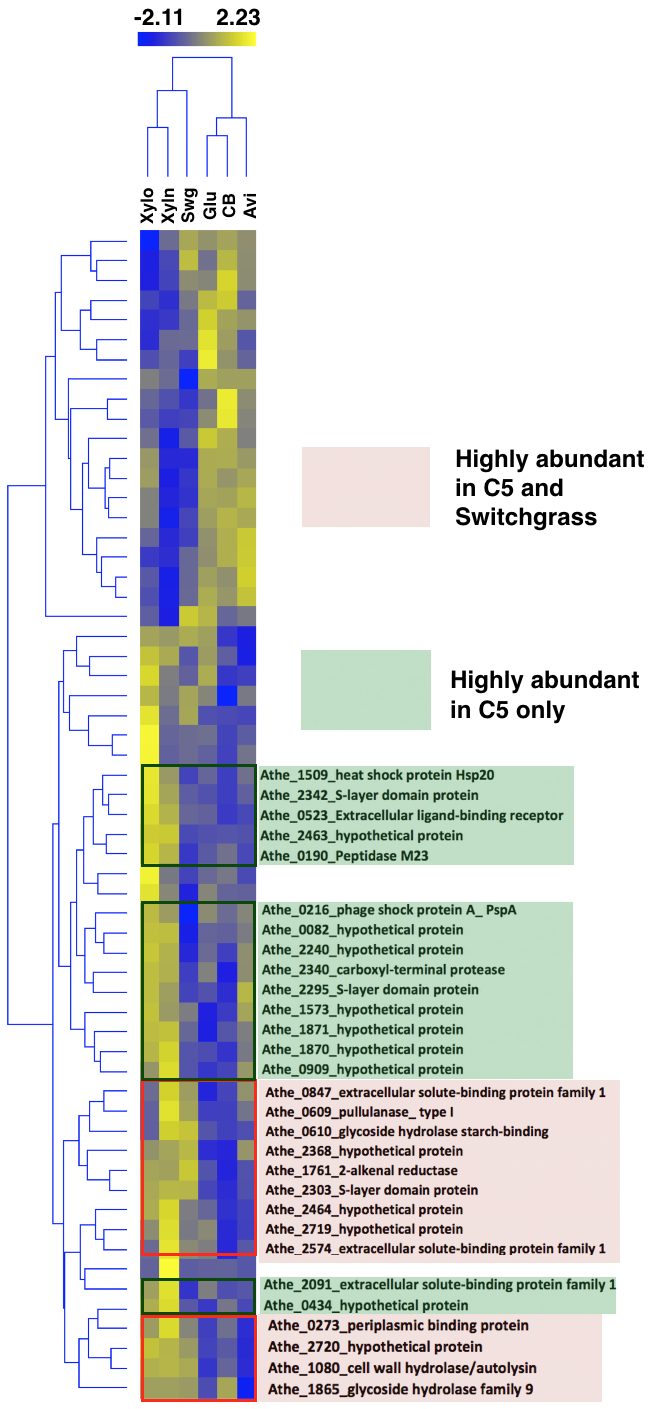


Fig. S4. **Heatmap of the abundances of proteins obtained by growing *C. bescii* in C5 substrate versus C6 substrates and on switchgrass (heterogeneous substrates consisting C5 and C6).** The red color indicates higher abundance in C5/Switchgrass and green color indicates higher abundance in C5 substrates only. Switchgrass is embedded between two different substrates (C5 and C6)**.**
